# Distinct Patterns of Evoked Firing in Engram and Non‐Engram Neurons in the Dentate Gyrus of Hippocampal Slices

**DOI:** 10.1002/hipo.70049

**Published:** 2025-11-14

**Authors:** Wen‐Chi Shu, Meyer B. Jackson

**Affiliations:** ^1^ Department of Neuroscience University of Wisconsin – Madison Madison Wisconsin USA; ^2^ Laboratory of Genome Integrity, National Cancer Institute, NIH Bethesda Maryland USA

**Keywords:** action potential bursts, dentate granule cell, immediate early genes, memory, novelty

## Abstract

Intense electrical activity modifies the cellular properties of neurons to enable the nervous system to store information. The dentate gyrus (DG) plays a role in learning and memory and its principal cell type, the granule cell (GC), can fire vigorously during behavior. To explore how experience modifies GC electrophysiology, we harnessed the immediate early gene, Fos, to target a genetically encoded hybrid voltage sensor to neurons that are activated by experience and are hypothesized to encode information. Voltage imaging from these engram GCs in mouse hippocampal slices revealed distinct patterns of stimulation‐evoked spiking that depended on prior experience. Compared to unchallenged mice, engram GCs from mice exposed to novelty burst more often, and have longer inter‐spike intervals following the blockade of inhibition. Voltage imaging from randomly targeted non‐engram GCs revealed distinct firing patterns that did not depend on novelty. Thus, experience modifies the firing of both engram and non‐engram GCs in distinctly different ways. The altered bursting will tune the facilitation of transmission from engram GCs to their various postsynaptic targets, and thus redirect the flow of information through the hippocampus. The pattern of GC firing constitutes a substrate for the encoding of information and will alter how the DG processes sensory inputs.

## 1 Introduction

Salient experiences modify the circuitry of the dentate gyrus (DG) to enable the storage of information. The principal neuron of the DG, the granule cell (GC), fires sparsely during most behaviors, but a small number (approximately 2%–4%) respond vigorously during exploration (Chawla et al. 2005; Hainmueller and Bartos 2018; Satvat et al. 2011). High‐frequency bursts of GCs are hypothesized to play roles in neural representation, and in the memory and retrieval of experience (GoodSmith et al. 2017; Jung and McNaughton 1993; Neunuebel and Knierim 2012; Senzai and Buzsáki 2017; Srinivasan et al. 2007; Vandael et al. 2020). Neurons contribute to information storage by changing their intrinsic properties and synaptic connections. Neurons that serve as substrates in the storage and recall of memories are commonly referred to as engram cells. (Choi and Kaang 2022; Guskjolen and Cembrowski 2023; Josselyn and Tonegawa 2020; Mayford 2014; Nambu et al. 2022; Schacter et al. 1978). Vigorous electrical activity triggers transcription of immediate early genes (IEGs), which investigators have exploited to label cells to study their roles in learning and memory (Barth et al. 2004; DeNardo and Luo 2017; Guzowski et al. 2001; Reijmers et al. 2007). IEG‐labeled cells form functional networks (Choi et al. 2018) and serve encoding roles in hippocampal place fields (Pettit et al. 2022). IEG‐labeled neurons can be activated to recapitulate encoding behaviors (Clawson et al. 2021; Cowansage et al. 2014; Liu et al. 2012; Ryan et al. 2015). Thus, IEG labeling offers a powerful approach to the targeting of behavior‐related neurons in order to study their role in learning and memory.

GCs synaptically excite CA3 pyramidal cells, hilar mossy cells, and various types of inhibitory interneurons. Bursting facilitates excitatory transmission to these postsynaptic targets and the strength of facilitation of each synapse is determined by the GC firing pattern (Geiger and Jonas 2000; Hashimotodani et al. 2017; Henze et al. 2002; McBain 2008; Mori et al. 2004; Mori et al. 2007). Thus, GC bursting plays a key role in the processing of cortical inputs by the DG. IEG activation during bursting promotes the production and accumulation of various molecules that strengthen and modify synapses (Dudek and Fields 1999; Paul et al. 2020; Roberts et al. 1998; Ryan et al. 2012). While recruited cells appear to have distinct intrinsic properties (Diamantaki et al. 2016; Pignatelli et al. 2019), and enhanced excitatory synaptic inputs (Sun et al. 2020), the functional properties of engram cells and the mechanisms by which they modify behavior remain unclear. These modifications in function outlast the experiences that induce them. The long‐term impact of experience on the spiking behavior of GCs is relevant to their roles in the encoding and storage of information, but little is known about how experience modifies the electrical activity of these neurons.

To address these questions we implemented a genetic strategy known as Targeted Recombination in Activated Populations (TRAP) (DeNardo and Luo 2017; Guenthner et al. 2013), and used it to drive expression of a genetically encoded hybrid voltage sensor (hVoS) (Chanda et al. 2005; Wang et al. 2010). This approach labeled cells based on activation of the IEG Fos, specifically capturing neurons that were activated during the first exposure to an experience. TRAP labeling targets neurons that can be activated optogenetically to recall experience (Allen et al. 2017; Clawson et al. 2021). We then used hVoS voltage imaging to investigate the stimulation‐evoked spiking behavior of GCs that had been activated during various forms of experience. We recorded evoked spikes in multiple GCs simultaneously in the DG of hippocampal slices (Shu and Jackson 2024), and observed distinct patterns of repetitive spiking in TRAP‐labeled engram GCs. The labeling of these neurons by an IEG, together with our observation of changes in firing properties meets two criteria for defining engram cells (Guskjolen and Cembrowski 2023): (1) They are activated during the initial experience, and (2) they exhibit persistent physiological changes. To compare, we also studied spiking in randomly targeted non‐engram Prox1‐labeled GCs, and observed that experience modified their firing differently from the modifications of TRAP‐labeled GC firing. The enhanced bursting and altered inter‐spike intervals of GCs will change how the DG processes inputs. The finding that IEG‐targeted and randomly targeted GCs change their firing properties in different ways indicates that experience results in encoding modifications of engram cells and global modifications of non‐engram cells.

## 2 Materials and Methods

### 2.1 Animal

Animal procedures have been approved by the Institutional Animal Care and Use Committee of the University of Wisconsin‐Madison (Protocol M005952). Double‐transgenic mice, Prox1::hVoS, TRAP1::hVoS, and TRAP2::hVoS, were generated by crossing the respective Cre drivers, Prox1‐CreER^T2^ (Prox1^tm3(cre/ERT2)Gco^, JAX, 022075) (Srinivasan et al. 2007), Fos^CreER^ (TRAP1, Fos ^tm1.1(cre/ERT2)Luo^, JAX, 021882) (Guenthner et al. 2013), and Fos^2A‐iCreER^ (TRAP2, Fos ^tm2.1(iCre/ERT2)Luo^, JAX, 030323) (Allen et al. 2017) with the Cre‐reporter Ai35‐hVoS1.5 mice (JAX, 031102) (Bayguinov et al. 2017).

### 2.2 Targeted Recombination in Active Populations

**FIGURE 1:**
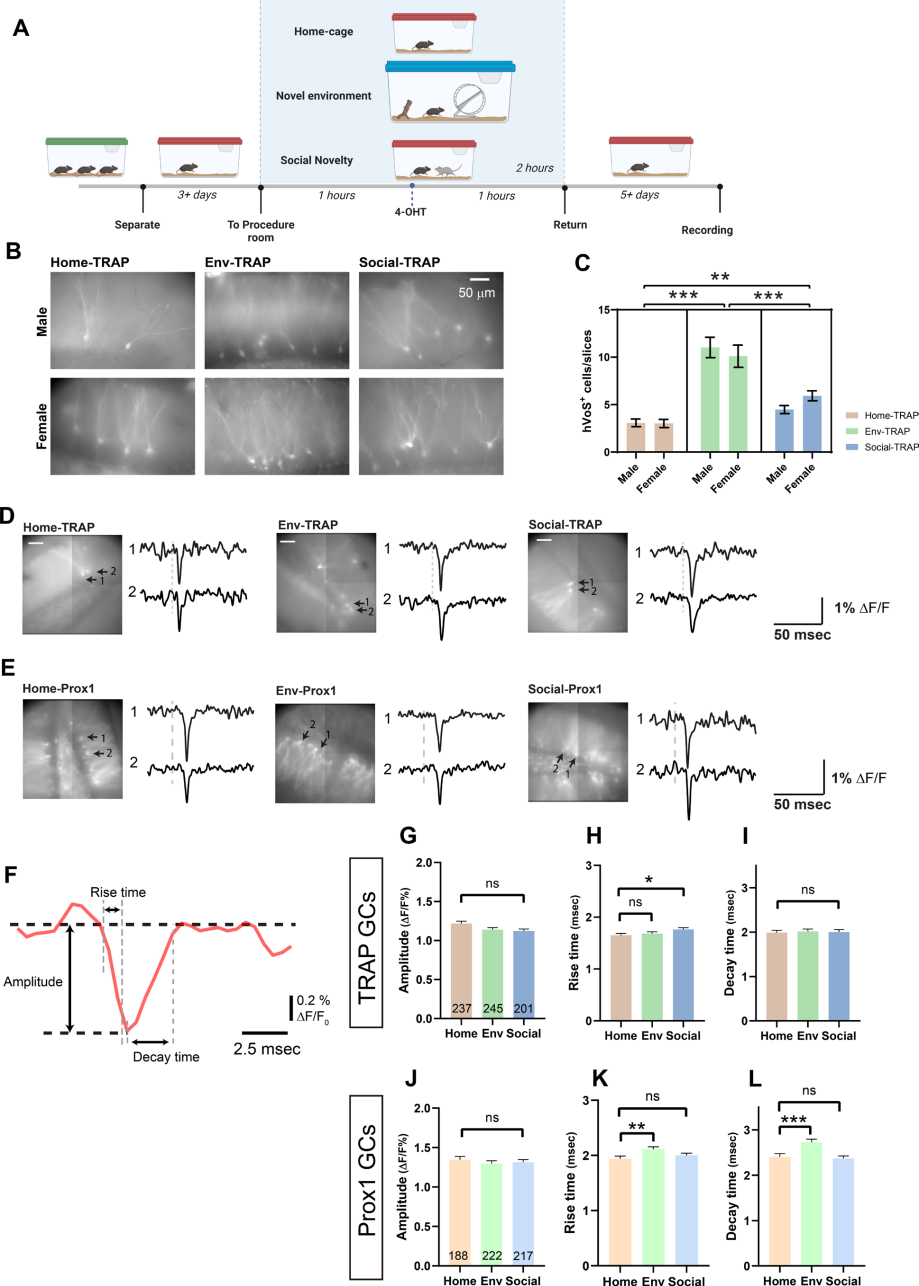
Labeling of GCs by experience and spike dynamics of labeled GCs. (A) Timelines for home‐cage, environment‐novelty, and social‐novelty paradigms. Mice were moved from standard housing to isolation for 3 or more days before spending 2 h in the indicated condition; 4‐OHT was administered at the first hour. Animals were returned to their home‐cage for 5–7 days before harvesting slices. (B) Fluorescence images display labeled GCs in each paradigm. (C) Numbers of labeled GCs ± SEM per slice in different paradigms. Slices from environment‐novelty (Env‐TRAP) and social novelty (Social‐TRAP) had more labeled cells (**p < 0.01; ***p < 0.001; two‐way ANOVA with Šídák's multiple comparisons test). (D) hVoS imaging of stimulus‐evoked voltage changes in slices from Home‐TRAP, Env‐TRAP, and Social‐TRAP mice, and (E) from Home‐Prox1, Env‐Prox1, and Social‐Prox1 mice. Traces are 5‐trial averages; Dashed lines: Time of 200 μA PP stimulation. Note that these images were from the lower‐resolution (80 × 80) high‐speed (2 kHz) CCD‐SMQ camera used for imaging as compared to the higher resolution images in B. (F) Fluorescent trace from a GC illustrating the measurement of spike parameters. (G–I) Spike properties of TRAP‐GCs for different experiences. Home‐TRAP: 117 slices, 32 mice; Env‐TRAP: 75 slices, 23 mice; Social‐TRAP: 83 slices, 16 mice. (G) Amplitudes (ΔF/F as %) were similar between different paradigms. (H) Rise‐time was longer in Social‐TRAP GCs compared to Home‐TRAP GCs. (I) Spike decay‐time did not differ significantly between the various experiences. (J–L) Spike properties of Prox1‐GCs for different experiences. Home‐Prox1: 24 slices, 3 mice; Env‐Prox1: 17 slices, 5 mice; Social‐Prox1: 28 slices, 3 mice. Cell numbers are indicated in the bars. (J) Amplitudes were similar between different paradigms. (K) Rise‐time was longer for Env‐Prox1 GCs. (L) Decay‐time was longer for Env‐Prox1 GCs (*p < 0.05; **p < 0.01, ***p < 0.001; One‐way ANOVA with Dunn's multiple comparisons test).

We employed TRAP1::hVoS (60 mice; 31 males, 29 females) and TRAP2::hVoS mice (50 mice; 22 males, 28 females) to target the hVoS 1.5 probe (Wang et al. 2010) to neurons based on their electrical activity during experiences. This study utilized three experience paradigms: a control paradigm leaving mice in their home cage (Home‐TRAP), an environmental novelty paradigm (Env‐TRAP), and a social novelty paradigm (Social‐TRAP). Each experimental procedure involved a 5–8 week‐old mouse of either sex, removed from its littermates and placed in a separate cage for at least 3 days. The mouse was then transferred to a procedure room to initiate the experience of novelty (see Figure 1A). Home‐TRAP mice (23 males, 25 females) remained in the procedure room in their original cages. Env‐TRAP mice (22 males, 24 females) were transferred into a larger cage with wood chunks, two igloos, and two running wheels. Social‐TRAP mice (8 males, 8 females) were given a new cage‐mate mouse of similar age and same sex. The Home‐cage condition involves movement of the cage, but because the mice remained in the same small cage without the many additions of environmental novelty and without a new cage‐mate, we refer to this condition as “no novelty.” One hour after transfer to the procedure room an intraperitoneal (IP) injection of 4‐hydroxytamoxifen (4‐OHT) was administered (H6278, Sigma‐Aldrich; 50 mg/kg in Chen oil; Chen oil, a 1:4 mixture of castor oil: sunflower seed oil; Sigma, Cat # 259,853 and S5007). The procedure continued for an additional hour after which the mouse was returned to the standard housing room for 5–7 days before being sacrificed for experiments.

### 2.3 Prox1‐GCs Labeling and Behavioral Paradigm

We used Prox1::hVoS animals to target the hVoS 1.5 probe to mature GCs (Shu and Jackson 2024). Tamoxifen (TAM, 30–80 μg per mouse, dissolved in 10/90 ethanol/sunflower oil, T5648, Sigma‐Aldrich) was orally administered during the first postnatal week to target developing GCs sparsely and randomly. Neonatally born GCs become functionally (Overstreet‐Wadiche et al. 2006) and morphologically (Kerloch et al. 2019) mature in ~2 weeks. This Prox1 procedure labels GCs that have mature morphology (Gupta et al. 2020), are located within the GC layer rather than the subgranular zone, and have matured well before the time of experimentation (at the age of 6–9 weeks). GCs thus labeled serve as a control for engram cells targeted by the TRAP system. Prox1::hVoS mice were also subjected to the same experiences as TRAP::hVoS mice (described above). They were individually housed for a minimum of 3 days and then moved to a procedure room for a total of 2 h. Home‐Prox1 mice remained in their home cage in the procedure room, Env‐Prox1 mice were placed in a novel environment, and Social‐Prox1 mice were given a new cage mate. Slices were harvested 5–7 days after returning the mice to standard housing. For comparison, we used Prox1::hVoS mice from standard housing with other mice and without novelty (Naïve‐Prox1) (Shu and Jackson 2024).

### 2.4 Hippocampal Slices Preparation

Hippocampal slices were prepared with a Vibratome (Leica VT 1200S) in ice‐cold, high‐magnesium, low‐calcium artificial cerebrospinal fluid (ACSF) composed of (in mM): 124 NaCl, 3.2 KCl, 1.25 NaH_2_PO_4_, 26 NaHCO_3_, 1 CaCl_2_, 6 MgSO_4_, and 10 Glucose (Ma et al. 2021). Slices 300 μm thick were cut in the coronal plane (approximately −1.3 to −2.6 mm relative to bregma) and allowed to recover in normal ACSF (as above but with 2.5 mM CaCl_2_ and 1.3 mM MgSO_4_) for 45 min at room temperature.

### 2.5 hVoS Imaging

Hippocampal slices were allowed to recover in normal ACSF containing 4 μM dipicrylamine (DPA) for 45 min at room temperature before voltage imaging. Slices were imaged with an upright epifluorescence Olympus BX51 microscope while perfusing with normal ACSF containing 4 μM DPA (Bayguinov et al. 2017; Ma et al. 2021). Images were acquired at 2 kHz with 80 × 80 resolution (336 × 336 μm^2^) using a high‐speed CCD‐SMQ camera (RedShirt/SciMeasure Imaging, Decatur GA). Acquisition, stimulation, and illumination were controlled by Neuroplex software provided with the camera, or by PhotoZ, an in‐house program (Chang 2006) adapted for use with this camera. Stimulation was initiated with computer interface‐triggered 200 μs current pulses to the PP with a stimulus isolator (ISO‐STIM‐01D, NPI Electronic, Tamm Germany) through an ACSF‐filled glass micropipette with a ~10 μm tip positioned in the middle or outer molecular layer. A 435 nm 29 W LED light source (Prizmatix) provided illumination through a 435 ± 10 nm bandpass filter (Chroma) and a CFP filter cube. High‐resolution brightfield and resting light images for evaluating location and morphology were acquired with a Kiralux CMOS Camera (Thorlabs CS505MU), coupled to the microscope with a sliding mirror. Gabazine (SR95531, Tocris Bioscience #1262) was diluted to a final concentration of 5 μM in freshly prepared DPA‐containing ACSF.

### 2.6 Data Analysis

**FIGURE 2:**
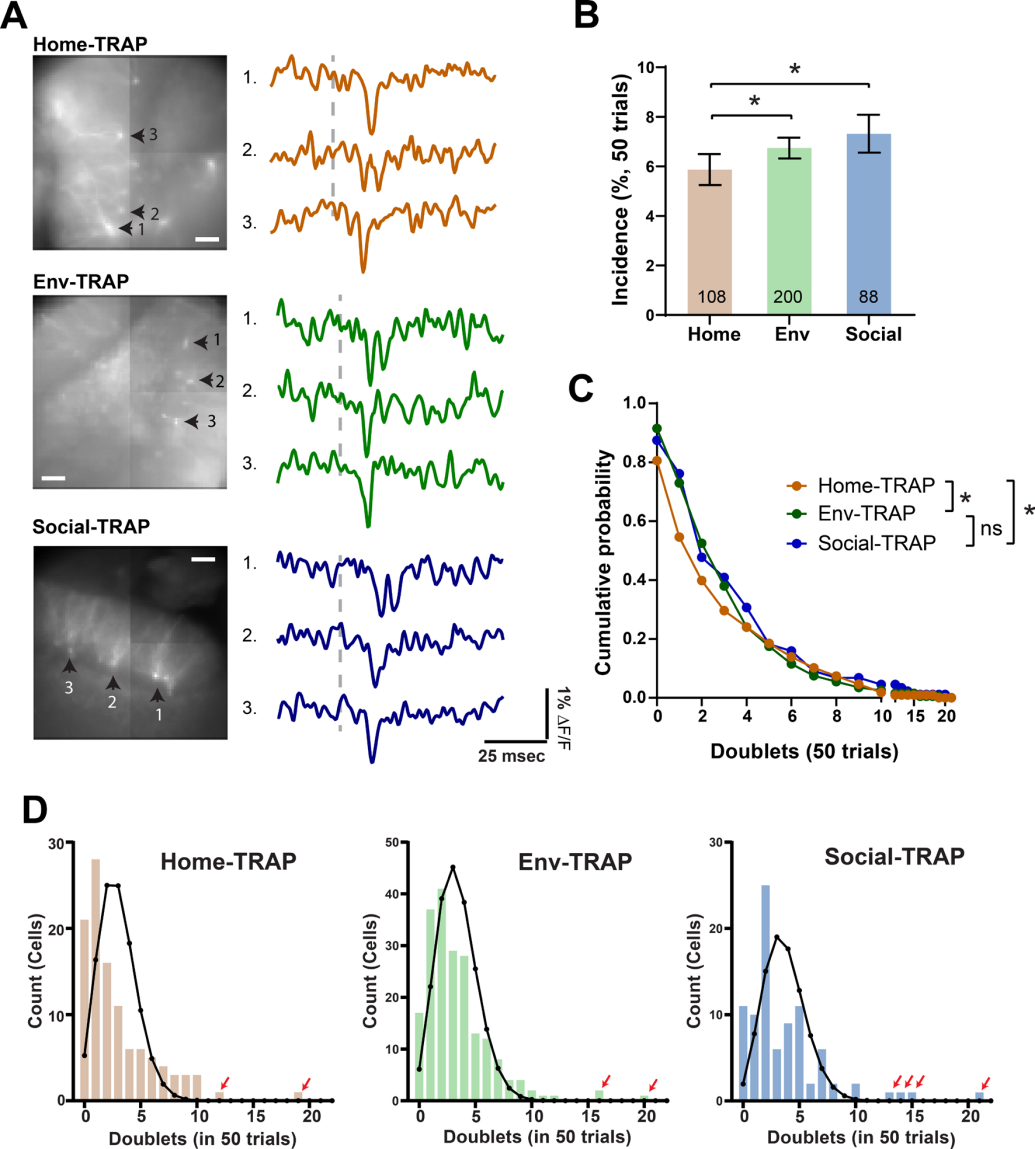
Doublet incidence in TRAP GCs. (A) Fluorescence images of slices indicating TRAP‐GCs with numbered arrows. GCs responded to PP stimulation with single spikes or doublets. Doublets are displayed by Cell #2 in Home‐TRAP, Cell #1 in Env‐TRAP, and Cell #1 in Social‐TRAP. Traces are single‐trial; vertical dashed lines indicate time of PP stimulation (200 μA). (B) In 50 trials, the mean doublet incidence ± SEM in Home‐TRAP GCs was lower than in Env‐TRAP GCs and Social‐TRAP GCs (*p < 0.05; Mann–Whitney *U*‐test). (C) Complementary cumulative distributions of GCs with a given number of trials with a doublet (*p < 0.05. Kolmogorov–Smirnov test). (D) Histograms and binomial fits reveal heterogeneity in doublet generation among TRAP‐GCs (Home‐TRAP: 35 slices, 12 mice; Env‐TRAP: 23 slices, 8 mice; Social‐TRAP: 39 slices, 9 mice; *p* < 0.0001, *χ* ^2^ test for all three groups using observed frequency compared to expected frequency). Red arrows indicate individual cells with incidence well outside the range.

**FIGURE 3:**
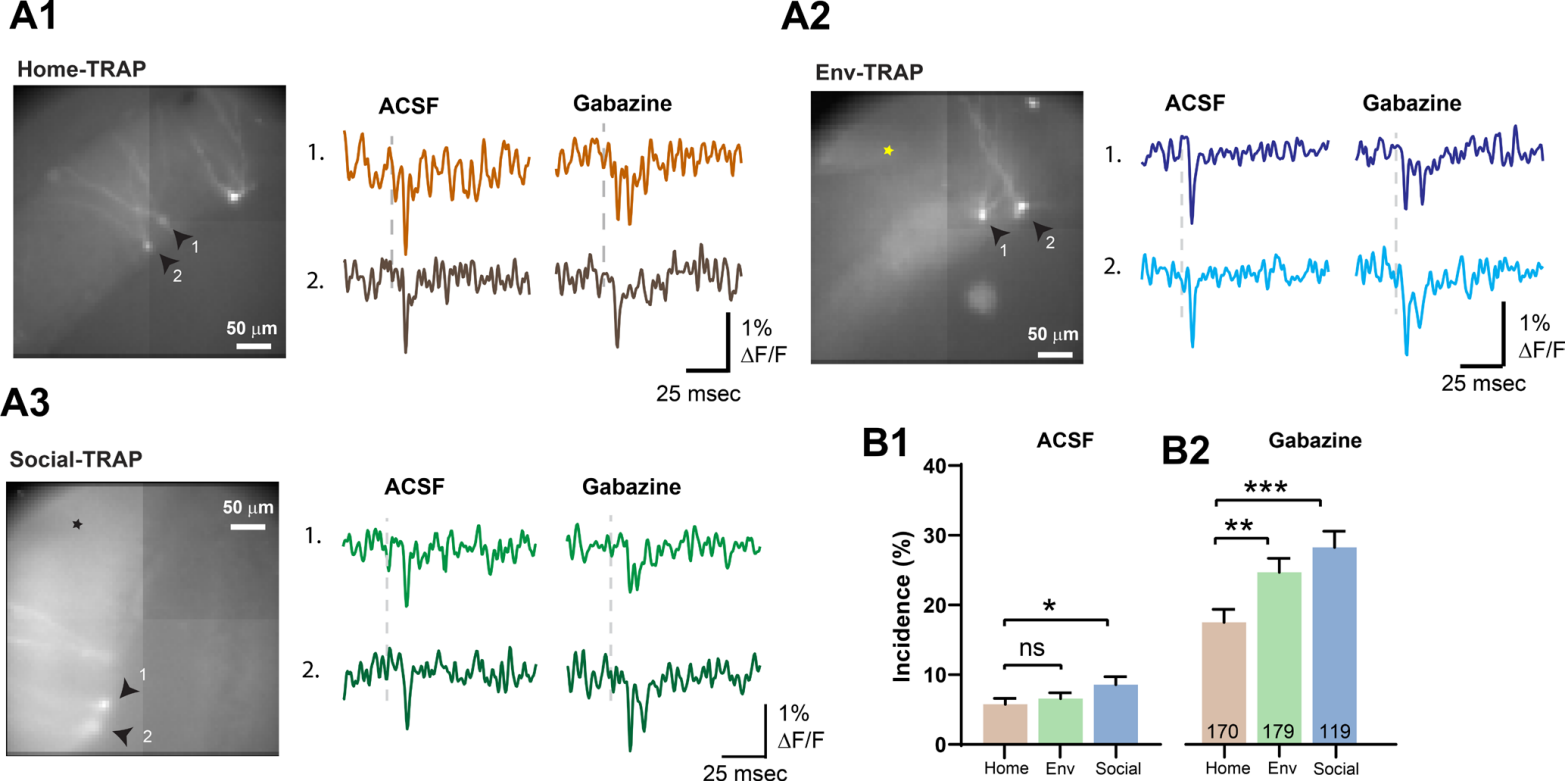
Doublets with blockade of inhibition. Fluorescence images from Home‐TRAP (A1), Env‐TRAP (A2) and Social‐TRAP (A3) slices. The site of stimulation is out of view in A1, marked by a yellow star in A2, and by a black star in A3. Selected GCs indicated in fluorescence images by numbered arrows. Traces (single‐trial) from these GCs show responses to PP stimulation with 200 μA, before and after adding 5 μM gabazine. (B) Doublet incidence ± SEM in Home‐TRAP, Env‐TRAP GCs, and Social‐TRAP GCs before (B1) and after (B2) gabazine addition. Doublet incidences in normal ACSF were similar to those with 50‐trials in Figure 2B, but here Home‐TRAP and Env‐TRAP are indistinguishable because the 5‐trial data has less statistical power. Incidences were all significantly longer in gabazine (*p < 0.05, **p < 0.01, ***p < 0.001; Mann–Whitney *U*‐test or paired *t*‐test; Home‐TRAP: 69 slices, 25 mice. Env‐TRAP: 57 slices, 30 mice, Social‐TRAP: 46 slices, 13 mice).

The first stage of analysis was performed using the data acquisition software. Spatial averaging was performed over small numbers of pixels within regions of interest containing GC somata. Probes expressing GCs were readily recognized by their location and characteristic dendritic arbors. Recorded fluorescence was divided by the resting light intensity to give ΔF/F, and low‐passed filtered with a 3‐point binomial filter. We applied a signal‐to‐noise ratio threshold of 4 to identify responsive cells. The mean signal‐to‐noise ratios were 12.66 ± 0.204 (*n* = 1076 GCs) and 9.21 ± 0.174 (*n* = 683 GCs) for Prox1 and TRAP, respectively. The fluorescence versus time traces from regions of interest were exported for further analysis and display. GC responses were analyzed in Igor 6 as described previously (Shu and Jackson 2024). Briefly, spikes were defined as events that had amplitudes (ΔF/F) exceeding 0.4% and could be fitted with an alpha function. This fitting process and subsequent spike measurements were based on single‐trial recordings. Spike amplitude is calculated as the height of the peak from the baseline (Figure 1F). The 10%–90% rise time is calculated as the time from 10% to 90% of peak amplitude. Spike decay time is measured by fitting the spike decay to an exponential. Traces displayed in Figures 2, 3, and 5 were single trials.

### 2.7 Experimental Design and Statistical Tests

Data were collected from 60 TRAP1::hVoS mice (31 males, 29 females), 50 TRAP2::hVoS mice (22 males, 28 females), and 38 Prox1::hVoS mice (18 males, 20 females). Littermates were randomly assigned to different groups (e.g., Home and Env) to avoid analysis bias due to genotypic background. A total of 2341 GCs were recorded, including 1248 TRAP‐GCs (416 Home‐TRAP from 187 slices, 46 mice; 537 Env‐TRAP from 137 slices, 46 mice; and 295 Social‐TRAP from 115 slices, 16 mice) and 1690 Prox‐GCs (437 Naïve‐Prox1 from 49 slices, 38 mice; 345 Home‐Prox1 from 30 slices, 3 mice; 491 Env‐Prox1 from 43 slices, 13 mice; and 417 Social‐Prox1 from 26 slices, 3 mice).

Statistical analysis and graphics were performed with GraphPad Prism 8. Normality was assessed for all datasets and paired *t*‐tests were applied to paired data that were normally distributed. When normality was not satisfied (most cases), nonparametric tests were used. For all multiple groups we initially applied the Kruskal–Wallis test (commonly referred to as one‐way ANOVA), followed by Dunn's multiple comparisons test when within‐group differences were significant. Two‐way ANOVA was followed by Sidak's post‐test when within‐group differences were significant. The Mann–Whitney *U*‐test was then used for direct pairwise comparisons. Distribution analyses included *χ* ^2^ tests for frequency comparisons and the Kolmogorov–Smirnov tests for cumulative distributions. k‐means clustering analysis was performed with RStudio and the number of clusters was estimated with the elbow method (Shu and Jackson 2024). Statistical tests are stated in the text and figure legends. Pharmacological experiments and 50‐trial recordings were performed on randomly selected slices.

## 3 Results

TRAP::hVoS mice, produced by crossing a TRAP Cre driver (Fos‐CreER^T2^) with the hVoS Cre reporter, target voltage probes to cells based on their electrical activity within a defined time window during which mice were presented with environmental novelty (Env‐TRAP), social novelty (Social‐TRAP), or no novelty (Home‐TRAP) (Figure 1A). After 5–7 days we prepared slices to assess labeling. Slices from Home‐TRAP mice had fewer labeled cells, but the numbers were sufficient to serve as a control to compare with Env‐TRAP and Social‐TRAP. As an activity‐independent control, we studied GCs labeled at random with Prox1‐CreER^T2^. In Prox1::hVoS mice, recombination was triggered by tamoxifen administered to neonatal mice to initiate hVoS probe expression early in development (Shu and Jackson 2024). Thus, labeling occurred at a few days of age long before the behavioral challenges. The Prox1‐labeled GCs are a small fraction drawn randomly from a large uniform population. If activity recruits TRAP neurons uniformly from the population of mature GCs, then Prox1::hVoS GCs and TRAP::hVoS GCs will have very little overlap (see Discussion). By the time of experiments at 6–10 weeks of age, neonatally born probe‐expressing Prox1 GCs have matured (Overstreet‐Wadiche et al. 2006; Kerloch et al. 2019). A low tamoxifen dose was used with Prox1::hVoS mice in order to label a small fraction of Prox1‐expressing cells. This yielded sparse labeling comparable to that in slices of TRAP::hVoS mice that experienced novelty. Sparse labeling (< 40 GCs in a 0.5 × 0.5 mm^2^ field of view) is essential for imaging electrical activity in single cells because at high density labeled cells overlap. Prox1::hVoS mice were subjected to the same experiences as TRAP::hVoS mice to produce Home‐Prox1, Env‐Prox1, and Social‐Prox1 mice. An additional group of Prox1::hVoS mice received a sham injection to determine the impact of separation and injection on Prox1 GC activity. Prox1::hVoS mice enabled us to assess the global impact of experience on randomly labeled GCs for comparison with engram GCs labeled with TRAP::hVoS mice.

We first assessed labeled‐cell density in TRAP::hVOS mice for different experiences, and determined action potential waveform by voltage imaging. We then turned our attention to the incidence of brief bursts of spikes and inter‐spike intervals, assessing the impact of experience first on TRAP::hVOS GCs and then on Prox1::hVOS GCs.

### 3.1 Behavior‐Dependent GC Targeting

Experience increased the number of labeled GCs in the DG of TRAP::hVoS mice. Following the time‐line in Figure 1A, mice were separated from their littermates and kept alone for at least 3 days before placing in a novel environment (Env‐TRAP), or adding a new mouse to their home cage (Social‐TRAP), or keeping in their home cage (Home‐TRAP). One hour after transfer, 4‐OHT was injected (labeling in TRAP::hVoS mice depended strictly on 4‐OHT injection). After another hour the mouse was returned to its home cage. After allowing 5–7 days for probe expression we prepared hippocampal slices. As reported previously with this protocol in TRAP mice, we observed labeled neurons in the DG (Guenthner et al. 2013). We cut slices in the coronal plane to obtain more dorsal/rostral DG, which has a higher density of Fos‐positive cells (Duffy et al. 2013).

TRAP labeling of GCs was seen in all behavioral paradigms (Figure 1B). Labeled GCs were easily distinguished by their location and morphology. We found that other types of neurons such as mossy cells and semilunar GCs were also labeled (Erwin et al. 2020), though with our protocols these cells were labeled less often than in previous reports. Semilunar GCs were usually located above the GC layer and had distinct morphological features that facilitate identification (Gupta et al. 2020), while mossy cells are located in the hilus, and are much larger with elaborate dendrites. Both of these cells were easily recognized and excluded from analysis. The average number of hVoS‐positive GCs per slice was 3.04 ± 0.31 in Home‐TRAP mice (3 males, 6 females), 10.61 ± 0.79 in Env‐TRAP mice (4 males, 4 females), and 5.16 ± 0.34 in Social‐TRAP mice (4 males, 3 females) (Figure 1C). Note that there are currently two TRAP mouse strains, TRAP1 and TRAP2, with different Cre insertion approaches (Allen et al. 2017; Guenthner et al. 2013). Our initial experiments employed TRAP1 and we subsequently switched to TRAP2, which does not disrupt endogenous Fos expression and uses a codon‐optimized Cre (DeNardo et al. 2019). Labeling of GCs with TRAP1 and TRAP2 was indistinguishable, as were plots of field synaptic potential and population spike amplitude versus stimulus current over the range 0–300 μA (Shu 2024). For these reasons TRAP1 and TRAP2 data were combined. Labeling differed significantly between all three behavioral paradigms, with the highest in Env‐TRAP and lowest in Home‐TRAP (Home‐TRAP versus Env‐TRAP, *p* < 0.001; Home‐TRAP versus Social‐TRAP, *p* = 0.0051; Env‐TRAP versus Social‐TRAP, *p* < 0.001, one‐way ANOVA with Holm‐Sidak's multiple comparison test). Both forms of novelty increased labeling significantly over Home‐TRAP to significantly different degrees.

The low labeling densities reflect the sparseness of Fos activation during behavior (Diamantaki et al. 2016). Social isolation can affect males and females differently (Guarnieri et al. 2020; Senst et al. 2016), and sex can influence exploration activity (Chen et al. 2021). Here, we observed no difference in the number of labeled GCs between sexes in any of the settings (Figure 1C, *F*(1,311) = 0.0614, *p* = 0.8044, two‐way ANOVA). The number of labeled GCs in female Social‐TRAP animals was slightly greater than in males, but the difference was not statistically significant (*p* = 0.4714). The labeling seen here with environmental novelty is consistent with reported increases in the number of Fos‐positive GCs in the dorsal DG (Duffy et al. 2013; Lüscher Dias et al. 2016; Temple et al. 2003). However, Fos immunoreactivity was previously reported not to be increased by social interaction (Lüscher Dias et al. 2016; von Heimendahl et al. 2012). These results are consistent with studies suggesting that the dorsal DG plays a greater role in spatial coding (Bannerman et al. 2002; Bannerman et al. 1999; Gilbert et al. 2001; Goodrich‐Hunsaker et al. 2008; Moser et al. 1995; Richmond et al. 1999). The lower but significant labeling of Social‐TRAP GCs indicates a role for the dorsal DG in social processing.

### 3.2 Impact of Experience on Spike Properties

The expression of the hVoS probe enabled us to use voltage imaging to evaluate the excitability of labeled cells. Because slices were prepared ~1 week after experience, these experiments focused on long‐lasting impacts. Simultaneous patch‐clamp recording has shown that the time course of hVoS probe fluorescence tracks action potentials with high temporal fidelity in single neurons (Bayguinov et al. 2017; Ghitani et al. 2015; Shu and Jackson 2024). This reflects the < 0.5 ms response time of hVoS fluorescence (Chanda et al. 2005). Electrical stimulation of the perforant path (PP) elicited spikes in multiple labeled GCs (Figure 1D,E), from which we determined spike amplitude, 10%–90% rise time, and decay time (Figure 1F). Spikes from TRAP GCs had similar amplitudes (Figure 1G, *p* = 0.0518, one‐way ANOVA) and decay times (Figure 1I, *p* = 0.8342, one‐way ANOVA) for all experiences. Social‐TRAP GC spikes had longer rise times (1.76 ± 0.037 ms, Figure 1H) compared to Home‐TRAP GCs (1.65 ± 0.035 ms; *p* = 0.0194, one‐way ANOVA with Dunn's multiple comparison test), which may reflect changes in sodium channel density or composition. But rise times were not significantly different between Home‐TRAP and Env‐TRAP GCs (1.69 ± 0.035 ms; *p* > 0.999, one‐way ANOVA with Dunn's multiple comparison test). Except for this small effect of social novelty on rise time, experience had no effect on the action potential waveform of GCs from TRAP mice that had different experiences.

Turning to Prox1 GCs, spike amplitudes were indistinguishable between Home‐Prox1, Env‐Prox1 and Social‐Prox1 GCs (*p* = 0.78, one‐way ANOVA; Figure 1J), as well as between uninjected and Sham‐injected Prox1 GCs (*p* = 0.1285, data not shown). However, spike rise times and decay times were significantly longer in Env‐Prox1 GCs than in Home‐Prox1 GCs (Figure 1K,L, *p* = 0.0014 for rise time, and *p* < 0.001 for decay time, one‐way ANOVA with Dunn's multiple comparison test). GCs from uninjected and Sham‐injected Prox1 mice had indistinguishable rise times (*p* = 0.759), but spike decay times were 13% longer in GCs from Sham‐injected Prox1 mice (*p* = 0.009; data not shown).

We compared the spikes between Prox1::hVoS and TRAP::hVoS mice subjected to the same experiences. Regardless of experience, TRAP GC spikes were faster, with briefer rise‐ and decay‐times than Prox1 GC spikes (*p* < 0.001 for both rise‐time and decay‐time, Prox1 versus TRAP GCs in home cage and both types of novelty, one‐way ANOVA with Dunn's multiple comparisons test). Thus, regardless of experience, Fos‐expressing GCs have faster spikes than randomly selected GCs. This may reflect preferential Fos expression in cells with faster spikes regardless of behavioral challenges.

### 3.3 Repetitive Firing of TRAP GCs and the Impact of Experience

A single pulse applied to the PP generally evokes a single spike in a GC. However, a small fraction of stimulations evoke brief bursts of two or more spikes (Dumenieu et al. 2018; Fricke and Prince 1984; Lynch et al. 2000), and this repetitive spiking is regulated by both intrinsic and network mechanisms (Shu and Jackson 2024). hVoS imaging reliably detected doublets, as verified with loose‐patch recording (Shu and Jackson 2024). Figure 2A displays traces of fluorescence versus time showing single and double spikes in Home‐TRAP GCs, Env‐TRAP GCs, and Social‐TRAP GCs. We previously showed that in coronal slices from Prox1 mice neonatally administered with tamoxifen from home cages with no novel experience (referred to here as Naïve‐Prox1 mice), GCs produce doublets at random over the course of 50 stimulations (Shu and Jackson 2024). Stimulation elicited doublets 5.7% ± 0.23% of the time, and the incidence in that study was well fitted by a binomial distribution indicating that in coronal slices mature GCs from naïve mice are homogeneous in their repetitive firing behavior. However, we show below that the incidence distribution for both Prox1 and TRAP‐labeled GCs deviates from binomial under different behavioral backgrounds.

Since repetitive firing impacts DG processing (Chamberland et al. 2018; Dumenieu et al. 2018; Henze et al. 2002; Mistry et al. 2011; Mori et al. 2007; Neubrandt et al. 2018), we investigated the effect of experience on doublets in TRAP‐labeled GCs. In 50 stimulations, we found that the incidence of doublets was 5.93% ± 0.42% in Home‐TRAP GCs (175 GCs, Figure 2B), which was similar to the previously reported value stated above for Naïve‐Prox1 GCs (Shu and Jackson 2024). Incidence differed between experience groups (*p* = 0.0386, Kruskal–Wallis test): Env‐TRAP GCs had a significantly higher incidence of 6.74% ± 0.42% (200 GCs, Figure 2B) compared to Home‐TRAP GCs (*p* = 0.017, Mann–Whitney test). The incidence in Social‐TRAP GCs of 7.318% ± 0.77% was even higher (88 GCs, *p* = 0.042, Mann–Whitney test), but incidences in Env‐TRAP GCs and Social‐TRAP GCs were indistinguishable (*p* = 0.8351, Mann–Whitney test). An increase in doublet incidence has the potential to facilitate transmission to postsynaptic targets, and we will expand on this point in the Discussion. The doublet incidence was generally lower in female mice (*F*(1,390) = 7.668, *p* = 0.0058, two‐way ANOVA), but sex did not interact with experience (*F*(2,390) = 1.747, *p* = 0.1757, two‐way ANOVA). Sex had no significant impact on doublet incidence in any group (Male–Female comparisons: Home‐TRAP, *p* = 0.9926; Env‐TRAP, *p* = 0.5188; Social‐TRAP, *p* = 0.9804; Sidak's multiple comparisons test), as seen previously in Naïve‐Prox1 GCs (Shu and Jackson 2024).

The cumulative distributions of doublet incidence differed between the paradigms tested here (Figure 2C, Home‐TRAP versus Env‐TRAP, *p* = 0.0176; Home‐TRAP versus Social‐TRAP, *p* = 0.0225; Env‐TRAP versus Social‐TRAP, *p* = 0.9748, Kolmogorov–Smirnov test). In contrast to the binomial doublet incidence distribution in Naïve‐Prox1 (Shu and Jackson 2024), the incidence distribution in all three groups of TRAP‐GCs deviated from binomial (Figure 2D), indicating that TRAP GCs are heterogeneous in their repetitive spiking behavior. Some TRAP GCs fired repetitively much more often than others (red arrows in Figure 2D). However, even when we excluded outliers using the interquartile range method, the distributions still deviated from binomial (Home‐TRAP, *χ* ^2^(8, *N* = 108) = 107.75, *p* < 0.001; Env‐TRAP, *χ* ^2^(8, *N* = 200) = 199.81, *p* < 0.001; Social‐TRAP, *χ* ^2^(10, *N* = 88) = 87.93, *p* < 0.001). This rules out a homogeneous population with a uniform doublet incidence, and indicates that GCs labeled by the TRAP protocol are heterogeneous in their firing properties.

Because inhibitory circuitry limits repetitive firing of GCs (Shu and Jackson 2024), we tested the action of the GABA_A_ receptor antagonist gabazine (Figure 3A). Here we stimulated with 5 trials instead of 50 trials (as in Figure 2). Changing solutions between control ACSF and gabazine requires ~5 min, so it was necessary to reduce the recording times in order to complete experiments within a period of time over which slice stability is maintained. In the absence of gabazine the incidence with 5 trials was similar to that seen with 50 trials. Social‐TRAP GCs had a mean incidence (9.08% ± 1.163%, 119 GCs) significantly higher than Home‐TRAP GCs (5.765% ± 0.84%, 170 GCs; *p* = 0.0495, Mann–Whitney test). Here the incidences of Env‐TRAP (6.592% ± 0.820%, 179 GCs) and Home‐TRAP GCs were indistinguishable in control ACSF (*p* = 0.3735, Mann–Whitney test), presumably due to the weaker statistical power of 5‐trial experiments. In gabazine, the differences were more robust. Gabazine increased the incidence 3–4 fold with values of 17.53% ± 1.85% in Home‐TRAP, 24.69% ± 2% in Env‐TRAP, and 28.24% ± 2.344% in Social‐TRAP (Figure 3B). These increases were all significant (*p* < 0.001 for all, Wilcoxon signed‐rank test). The incidence differed between experience groups (*p* = 0.0001, Kruskal–Wallis test): incidences in Env‐TRAP GCs and Social‐TRAP GCs were significantly higher than in Home‐TRAP GCs (*p* = 0.0086 and *p* < 0.001, respectively. Mann–Whitney test). The higher incidence of doublets in Env‐TRAP and Social‐TRAP compared to Home‐TRAP suggests that both forms of novelty enhance repetitive firing of GCs. Two‐way ANOVA on the data in Figs. 3B1 and 3B2 indicated highly significant effects of gabazine (*p* < 0.0001) and behavior (*p* = 0.0002), and further indicated a significant interaction (*p* = 0.0314). The interaction indicates that the effects of behavior can be strongly amplified by limiting inhibition.

### 3.4 Impact of Experience on Inter‐Spike Intervals in TRAP‐GCs

**FIGURE 4:**
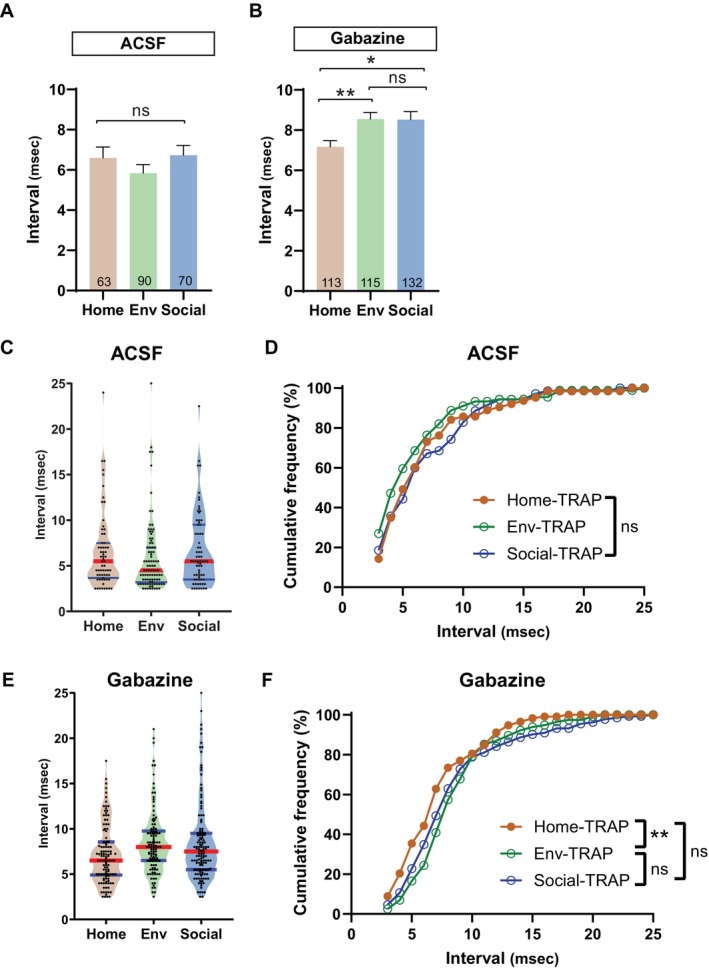
Doublet inter‐spike intervals in TRAP GCs. (A) In ACSF, experience had no significant impact on mean doublet interval. (B) In gabazine, intervals were longer in both Env‐TRAP and Social‐TRAP versus Home‐TRAP GCs (*p < 0.05; **p < 0.01; Mann–Whitney test). (C) Violin plots of intervals, median (red), and quartiles (blue) in ACSF. (D) Cumulative frequency distributions illustrate the distribution of inter‐spike intervals recorded from GCs in ACSF, with no significant differences across conditions (Kolmogorov–Smirnov test). (E) Violin plots of intervals in gabazine. (F) Cumulative frequency distributions of inter‐spike intervals in gabazine. Environmental‐novelty results in a significantly different interval distribution compared to home‐cage. The social‐novelty distribution was indistinguishable from both the home‐cage and environmental novelty distributions (**p < 0.01; Kolmogorov–Smirnov test).

Because synaptic facilitation is generally a sensitive function of inter‐spike interval (Jackman and Regehr 2017; Katz and Miledi 1968; Neubrandt et al. 2018), we evaluated the time intervals between the first and second spikes of doublets. No significant effect of experience on inter‐spike interval could be seen in control ACSF (Figure 4A1, *p* = 0.1683, one‐way ANOVA), but gabazine unmasked differences (Figure 4A2, *p* = 0.0036, one‐way ANOVA). Gabazine prolonged intervals in Env‐TRAP and Social‐TRAP GCs (*p* < 0.001) but not in Home‐TRAP GCs (*p* = 0.1481, Mann–Whitney test). In gabazine, Home‐TRAP GC doublets had a mean interval of 7.17 ± 0.307 ms, which was significantly shorter than Env‐TRAP GCs (8.55 ± 0.336 ms; *p* = 0.001) and Social‐TRAP GCs (8.52 ± 0.395 ms; *p* = 0.0244, Mann–Whitney test for all). Thus, both forms of novelty prolonged the inter‐spike interval.

The inter‐spike intervals of doublets recorded from TRAP‐GCs exhibit a skewed, multimodal distribution, as illustrated with violin plots (Figure 4C,E; medians indicated with red lines and quartiles with blue lines). k‐means analysis suggested that intervals fell into three clusters in all groups, with similar but not identical boundaries. This clustering indicates that doublets fall into subgroups with distinct intervals, and suggests that GCs are heterogeneous in the circuit and intrinsic properties that generate multiple spikes. To determine whether experience alters the interval distribution, we compared the cumulative frequencies using the two‐sample Kolmogorov–Smirnov test (Figure 4D,F). In ACSF, the distribution of intervals was not impacted by novelty (Home‐Env: *p* = 0.473, Home‐Social: *p* = 0.905, Env‐Social: *p* = 0.153), recapitulating the comparison of mean intervals (Figure 4A). In gabazine, the distributions of inter‐spike intervals differed between Home‐TRAP and Env‐TRAP GCs (*p* = 0.0032), again recapitulating the comparison of means (Figure 4B). Experience shifted the distributions toward longer intervals. Thus, blockade of inhibition brings out a more nuanced effect of experience on spike timing in TRAP GC doublets.

### 3.5 Impact of Experience on Randomly Targeted GCs

**FIGURE 5:**
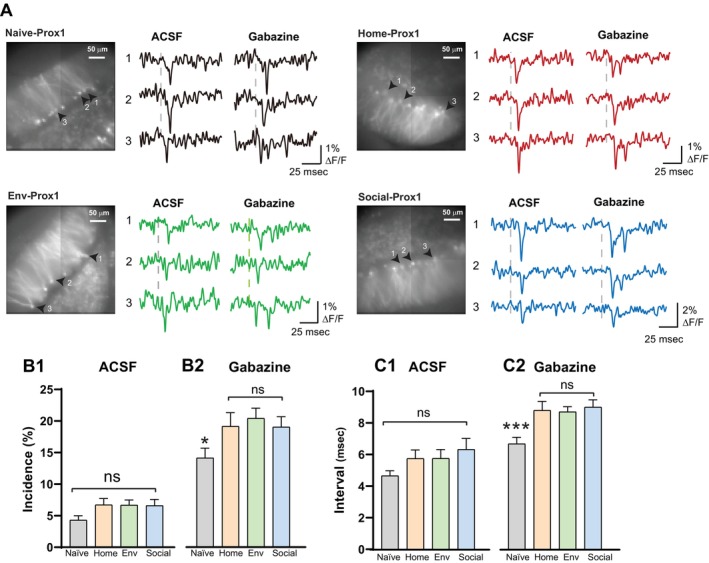
Impact of experience on doublet incidence and interval in Prox1 GCs. (A) Fluorescence images of slices from Naïve‐Prox1, Home‐Prox1, Env‐Prox1, and Social‐Prox1 mice, with selected GCs indicated by numbered arrows. Traces (single‐trial) show responses to PP stimulation (200 μA) before and after addition of 5 μM gabazine. (B1) In ACSF without gabazine, incidences are indistinguishable between conditions. (B2) In gabazine, all forms of experience, including home‐cage and novelty, resulted in higher doublet incidence compared to GCs from Naïve‐Prox1 mice, while no significant differences were observed between GCs from Home‐, Env‐, and Social‐Prox1 mice. (Naïve‐Prox1 GCs: *N* = 219 cells, 30 slices, 19 mice; Home‐Prox1: *N* = 143 cells, 15 slices, 3 mice; Env‐Prox1: *N* = 231 cells, 16 slices, 14 mice; Social‐Prox1: *N* = 170 cells, 19 slices, 3 mice. *, *p* < 0.05; one‐way ANOVA with Dunn's multiple comparisons test, F(3, 759) = 14.32, *p* = 0.0025; F(2, 541) = 1.056, *p* = 0.5897). (C1) Doublet intervals were indistinguishable in ACSF without gabazine. (C2) In gabazine, all experiences significantly prolonged the interval compared to Naïve‐Prox1 (*F*(3, 379) = 20.32, *p* = 0.007; *F*(2, 292) = 0.1988, *p* = 0.9054).

Our experiments on slices from TRAP mice showed that novelty alters the firing properties of engram GCs, thus implying that repetitive firing has a role in encoding. However, it is possible these changes reflect a global impact of experience on the DG that modifies GCs independent of their specific encoding functions. We therefore investigated the impact of experience on GC firing properties in slices from Prox1::hVoS mice, where targeting occurs at random following neonatal tamoxifen administration long before the behavioral challenges. These mice were subjected to the same experiences as TRAP::hVoS mice (Figure 1A), but with no 4‐OHT injection during the experience. In these comparisons, we also included Naïve‐Prox1 GCs and Sham‐injected‐Prox1 GCs. The Naïve‐Prox1 GCs were from control mice maintained in standard housing together with littermates, while Sham‐injected‐Prox1 GCs were from mice separated from their littermates for a week and injected with vehicle. (Note that the uninjected condition cannot be tested in TRAP mice because without injection no cells are labeled). Voltage imaging before and after adding gabazine revealed stimulation‐evoked spikes and doublets in GCs from Prox1::hVoS mice that had various experiences (Figure 5A). We did not observe significant differences in doublet incidence or interval between Naïve‐Prox1 and Sham‐injected‐Prox1 GCs in control ACSF (Incidence: *p* = 0.4144; Interval: *p* = 0.1266; Mann–Whitney test) or ACSF with gabazine (Incidence: *p* = 0.6675; Interval: *p* = 0.0628; Mann–Whitney test). These results suggest that separation and injection do not have a detectable effect on GC repetitive firing. Without blockade of inhibition, the mean doublet incidence was indistinguishable between Naïve‐Prox1 (4.29% ± 0.703%, 219 GCs), Home‐Prox1 (6.71% ± 1.011%, 143 GCs), Env‐Prox1 (6.67% ± 0.82%, 231 GCs), and Social‐Prox1 (6.59% ± 0.984%, 170 GCs; Figure 5B1, *p* = 0.0898, one‐way ANOVA). Gabazine increased the doublet incidence, and a triplet (three spikes) can be seen in cell #3 from the Naïve‐Prox1 slice in Figure 5A. (Triplets were rarer and although their incidence has been quantified previously (Shu and Jackson 2024), the numbers are generally too low to be useful in assessing experience). In gabazine, the doublet incidence varied with condition (Figure 5B2, *F*(3, 759) = 14.32, *p* = 0.0025, one‐way ANOVA), and in Naïve‐Prox1 (14.16% ± 1.53%) the incidence was significantly lower than in Home‐Prox1 (19.16% ± 2.155%, *p* = 0.048, Mann–Whitney test), Env‐Prox1 (20.43% ± 1.59%, *p* = 0.0008, Mann–Whitney test), and Social‐Prox1 (19.06% ± 1.625%, *p* = 0.0012, Mann–Whitney test). However, there were no significant differences between the doublet incidences in the three experience conditions (*F*(2, 541) = 1.056, *p* = 0.5897, one‐way ANOVA). The higher incidence of doublets in these conditions (Home, Env, and Social) compared to Naïve indicates that the experience of separation and transfer to the procedure room impacted the firing properties of randomly‐targeted GCs regardless of whether or not the environment had additional novel features. Thus, in contrast to TRAP GCs, novelty had no effect on Prox1 GCs.

The doublet intervals of Prox1 GCs were indistinguishable in the absence of gabazine (Figure 5C1; *p* = 0.2532, one‐way ANOVA). As seen previously (Shu and Jackson 2024), gabazine prolonged the doublet interval (Figure 5C2; *p* = 0.0001, one‐way ANOVA) to 6.68 ± 0.408 ms in Naïve‐Prox1 GCs (Shu and Jackson 2024). Intervals were significantly longer in Home‐Prox1 GCs (8.79 ± 0.570 ms, *p* < 0.0001), Env‐Prox1 GCs (8.704 ± 0.328 ms, *p* < 0.0001), and Social‐Prox1 GCs (9.00 ± 0.454 ms, *p* = 0.0002, Mann–Whitney test). Thus, as shown above for incidence, the environment had an impact on Prox1 GC doublet intervals, but the degree of novelty did not.

**FIGURE 6:**
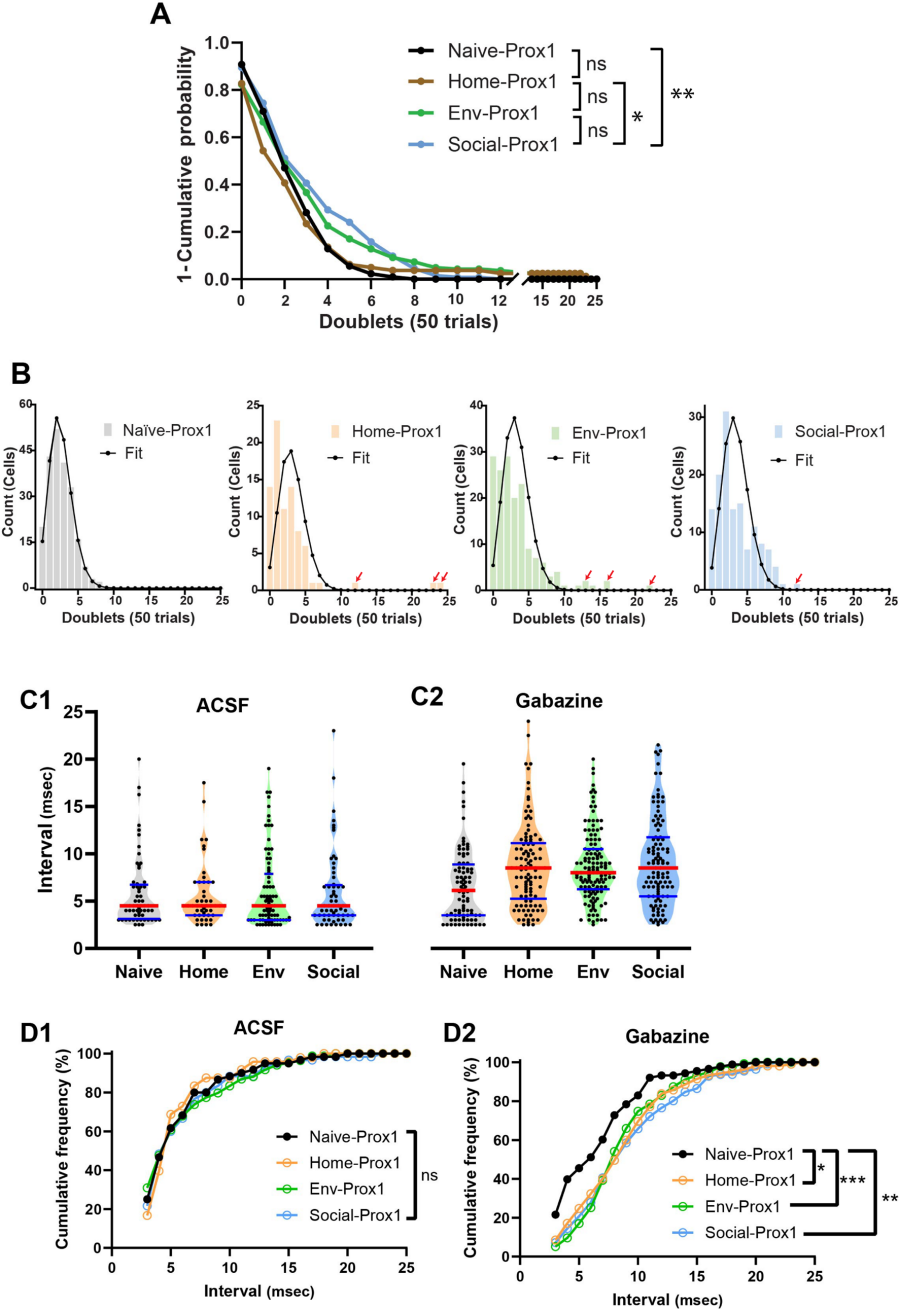
Doublet incidence and interval distributions in Prox1 GCs. (A) Complementary cumulative distributions of GCs with a given number of trials with a doublet (*, *p* < 0.05; **p < 0.01, Kolmogorov–Smirnov test; other distributions were indistinguishable, see text). (B) Histograms and binomial fits reveal heterogeneity in doublet generation among Prox1‐GCs (Naïve‐Prox1, 218 cells, 19 slices, 6 mice; Home‐Prox1, 82 cells, 10 slices, 3 mice; Env‐Prox1, 164 cells, 13 slices, 3 mice; Social‐Prox1, 133 cells, 13 slices, 3 mice). Red arrows indicate cells with incidence well outside the range. (C) Violin plots of intervals with median (red) and quartiles (blue) in ACSF (C1) and gabazine (C2). (D1) Cumulative frequency distribution of inter‐spike intervals in Prox1‐GCs in normal ACSF. There were no significant differences in interval distribution between groups (Naïve vs. Home, *p* = 0.9926, Naïve vs. Env, *p* = 0.9235, Naïve vs. Social, *p* = 0.9251, Home vs. Env, *p* = 0.5612, Home vs. Social, *p* = 0.9130, and Env vs. Social, *p* = 0.9235, Kolmogorov–Smirnov test). (D2) Inter‐spike interval distributions in gabazine indicated that experience has an impact (**p < 0.01; ***p < 0.001, Kolmogorov–Smirnov test).

We then examined the doublet incidence distribution of Prox1 GCs. Cumulative incidence distributions are presented in Figure 6A (the Naïve‐Prox1 distributions are reproduced from the coronal slice distributions in fig. 4 of Shu and Jackson (2024) in order to compare with the present Prox1 GC data). The cumulative distributions of Prox1 incidence differed between the paradigms tested, and this dependence on paradigm reflected the difference between Home‐Prox1 and Social‐Prox1 and between Naïve‐Prox1 and Social‐Prox1 (Figure 6A; Naïve‐Prox1 vs. Home‐Prox1, *p =* 0.0918; Naïve‐Prox1 vs. Env‐Prox1, *p =* 0.1659; Naïve‐Prox1 vs. Social‐Prox1, *p =* 0.0069; Home‐Prox1 vs. Env‐Prox1, *p* = 0.3904; Home‐Prox1 vs. Social‐Prox1, *p* = 0.0413; Env‐Prox1 vs. Social‐Prox1, *p* = 0.7389; Kolmogorov–Smirnov test). In GCs from Naïve‐Prox1 mice, the distribution was consistent with a binomial distribution (Figure 6B), but the Home‐Prox1, Env‐Prox1, and Social‐Prox1 distributions deviated from binomial. As noted in the TRAP‐GC distributions (Figure 2D), with Prox1 GCs, some GCs generated doublets with a markedly higher frequency (red arrows in Figure 6B). Even after removing outliers using the interquartile range method, the distributions remained significantly non‐binomial (Home‐Prox1: *χ* ^2^ (7, *N* = 82) = 84.71, *p* < 0.001; Env‐Prox1: *χ* ^2^(8, *N* = 164) = 87.56, *p* < 0.001; Social‐Prox1: *χ* ^2^(10, *N* = 133) = 87.96, *p* < 0.001). These results indicate that experience modifies the firing properties of randomly targeted GCs nonuniformly.

We then examined the distribution of intervals using violin and cumulative frequency plots. k‐means analysis again indicated 3 clusters of intervals in Prox1 GCs. Distributions were multimodal and skewed (Figure 6C1,C2,D1,D2), and qualitatively similar to those in TRAP GCs (Figure 4C–F). In control ACSF, the cumulative frequency distributions were similar regardless of behavioral challenge (Figure 6D1, Naïve‐Prox1 versus Home‐Prox1: *p* = 0.9926, Naïve‐Prox1 versus Env‐Prox1: *p* = 0.9235, Naïve‐Prox1 versus Social‐Prox1: *p* = 0.5612; Kolmogorov–Smirnov tests). However, the application of gabazine brought out differences (Figure 6D2). Doublets in Naïve‐Prox1 GCs had a larger proportion of shorter intervals, and the distribution differed significantly from Home‐Prox1 (*p* = 0.0015), Env‐Prox1 (*p* = 0.0001), and Social‐Prox1 (*p* = 0.0026). In gabazine, Home‐Prox1 mice had cumulative interval distributions indistinguishable from Env‐Prox1 mice (*p* = 0.4718) and Social‐Prox1 mice (*p* = 0.937). In summary, except for the different incidence distributions between Naïve and Home versus Social‐Prox1, randomly targeted Prox1 GCs displayed similar firing behavior in ACSF between behavioral conditions. In gabazine, experiencing a different environment altered both the incidence and interval of doublets, but the novelty of that environment was not a factor. Overall, the impacts of environment were less pronounced in randomly targeted Prox1 GCs than in engram TRAP GCs. But changes in environment, with or without novelty, resulted in GCs with more doublets, altered incidence distributions, and longer inter‐spike intervals.

### 3.6 Comparison Between TRAP GCs and Prox1 GCs

**FIGURE 7:**
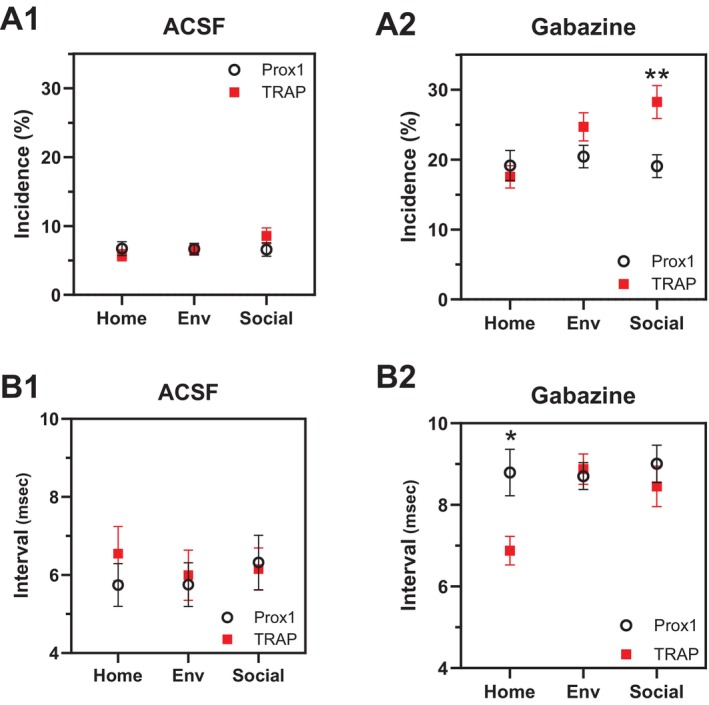
Comparing the impact of experience between Prox1 and TRAP GCs. Comparisons of doublet incidence and interval with and without gabazine. (A1) Doublet incidence for home‐cage, environmental‐novelty, and social‐novelty in ACSF. Results did not depend on experience (*p* = 0.3775, two‐way ANOVA), genotype (*p* = 0.6769), or their interaction (*p* = 0.3135). (A2) Doublet incidence in gabazine. Results depended on experience (*p* = 0.0156, two‐way ANOVA), genotype (*p* = 0.0109), and their interaction (*p* = 0.0239), with a significant difference seen with social novelty (**, *p* = 0.0044, Sidak's post hoc test). (B1) Doublet interval in ACSF. Results did not depend on experience (*p* = 0.8197, two‐way ANOVA), genotype (*p* = 0.5676), or their interaction (*p* = 0.7576). (B2) Doublet interval in gabazine. Results depended on genotype (*p* = 0.0286) and showed a near‐significant effect of experience (*p* = 0.057; two‐way ANOVA), and interaction (*p* = 0.0509), with a significant difference with Home (*p = 0.0117, Sidak's post hoc test).

Data from activity‐targeted TRAP GCs and randomly‐targeted Prox1 GCs provide an opportunity to compare the effect of experience between encoding and non‐encoding neurons. The small fraction of GCs, 2%–4%, that express Fos during experiences such as those tested here (Chawla et al. 2005; Satvat et al. 2011; Schmidt et al. 2012), as well as our own observations of low labeling densities (Figure 1C), ensure that we have minimal overlap between neurons labeled by the two approaches (see Section 4). We therefore compared spike firing between TRAP GCs and Prox1 GCs. The incidence did not depend on genotype when recordings were performed in ACSF (Figure 7A1; *p* = 0.6769, two‐way ANOVA), but genotype had an impact in gabazine (Figure 7A2, *p* = 0.0109), with Social‐TRAP GCs having a significantly higher incidence than Social‐Prox1 GCs (*p* = 0.0044, Sidak's post hoc test). This represents a difference in incidence between engram and non‐engram cells.

Turning to doublet intervals, TRAP GCs and Prox1 GCs were indistinguishable in ACSF (Figure 7B1, *p* = 0.5676, two‐way ANOVA), but in gabazine genotype had an impact (Figure 7B2, *p* = 0.0286), and Home‐Prox1 GCs had longer intervals than Home‐TRAP GCs (*p* = 0.0117, Sidak's post hoc test). Thus, in both engram and non‐engram GCs, doublet incidence and interval were modified by experience, but the modifications differed. The differences could only be seen when inhibition was blocked, and were expressed under limited behavioral conditions.

## 4 Discussion

This study has evaluated the impact of experience on stimulation‐induced firing in two distinct populations of GCs. The TRAP‐targeted cells encode the memory of an animal's initial experience of a novel environment or of a novel conspecific. These GCs expressed a voltage sensor after their electrical activity rose to a level sufficient to initiate Fos expression during a behavioral challenge. The Prox1 mouse targeted GCs randomly at an early age long before the behavioral challenge. To assess potential overlap between these two populations, we estimate that one slice from a Prox1 mouse has ~60 labeled GCs, including GCs near and below the surface. This sparse labeling reflects our low dose of TAM to induce Cre‐dependent recombination in a very small fraction of the large number of Prox1‐expressing GCs. Likewise, we estimate ~15 labeled cells per slice in TRAP mice (extending the value in Figure 1C to include cells below the surface). With 5 × 10^5^ GCs in the mouse DG (Bonthius et al. 2004), a 300 μm slice contains ~15,000 GCs. Assuming that TRAP and Prox1 cells are each drawn randomly from the total, we can estimate that ~0.1% of labeled GCs in a Prox1 slice are engram cells and 0.4% of engram cells would be labeled from a Prox1 slice. Thus, there is no significant overlap, and we can compare TRAP GCs with Prox1 GCs to distinguish between encoding changes and global changes in a population of GCs that were either quiescent or had activity below the threshold for Fos activation during the experiences. Experience altered the spiking characteristics of each population of GCs in different ways, and the nature of the experience made a difference. We found that spike amplitude is insensitive and invariant under all conditions (Figure 1G,H). Rise times and decay times were generally briefer in TRAP GCs than Prox1 GCs under all conditions, and experience had almost no impact. The greater complexity of doublets proved to be a richer source of information. The incidence of these brief bursts as well as their inter‐spike intervals varied between behaviors in both engram GCs and non‐engram GCs. The variations in these aspects of spiking suggest that experience can modify the function of GCs to alter how the DG processes sensory input.

TRAP GCs labeled by environmental novelty and social novelty had higher doublet incidences (Figure 3A). The differences between TRAP and Prox1 (Figure 7) suggest either selective recruitment of a pre‐existing population of more strongly bursting GCs, or modifications in GC bursting during experience. Blockade of GABA_A_ receptors amplified the differences in spike incidence between TRAP GCs for different experiences (Figure 3B1,B2), but even in gabazine novelty had no effect on Prox1 GCs (Figure 5B2). This suggests that novelty alters inhibitory inputs to engram cells, as has been seen in the CA1 region (Yap et al. 2021).

Inter‐spike intervals were also impacted by experience. In the presence of gabazine, TRAP GCs from mice that had experienced novelty had doublets with longer intervals (Figure 4B). Prox1‐GCs from the Home, Env, and Social groups all showed longer intervals in the presence of gabazine compared to naïve controls (Figure 5C2), suggesting that any environmental transition is sufficient to alter the firing in this GC population. Gabazine did not prolong intervals in Home‐TRAP GCs suggesting that inhibition has less of a role in controlling inter‐spike intervals in these cells. Furthermore, environmental novelty shifted the distribution of intervals in TRAP GCs when inhibition was blocked, but not with Social‐TRAP and Home‐TRAP (Figure 4F).

The changes in TRAP and Prox1 GC doublets indicate that experience modifies the properties of both engram and non‐engram GCs. Given the extremely low likelihood that a Prox1‐targeted GC is recruited by activity to become an engram GC (0.1% estimated above), the changes in Prox1 GCs indicate that GCs that remain quiescent, or are weakly active below the IEG threshold during an experience, are still modified as part of a global response. The modifications in Prox1 GCs reported here are reminiscent of the global modifications in pyramidal cell action potentials observed in learning paradigms (Disterhoft and Oh 2006). Changes in neurons not directly engaged in encoding during experience may reflect homeostatic plasticity as the entire network adapts (Turrigiano 2012). The observation of changes in both TRAP GCs and Prox1 GCs underscores the need to use complementary labeling strategies to assess the impact of experience on network function. Comparisons between these two populations of GCs enable the assignment of modifications to different encoding functions. Behaviors activate other IEGs (DeNardo and Luo 2017; Flavell and Greenberg 2008; Yap and Greenberg 2018), and voltage imaging experiments harnessing these expression vehicles can be expected to target intersecting populations of cells to reveal additional functional distinctions. Further experiments with various IEG labeling strategies, as well as more detailed studies of how electrical activity stimulates the expression of IEGs, will deepen our understanding of the relation between activity and behavior, and the representation of sensory inputs by neural circuits.

In a GC successive spikes are triggered by both intrinsic excitability and recurrent excitation, and are restrained by synaptic inhibition (Shu and Jackson 2024). Low‐threshold T‐type calcium channels modulate GC burst firing, regulate transmission to the CA3 region (Dumenieu et al. 2018), and promote GC long‐term synaptic plasticity (Kennedy et al. 2024). G‐protein modulation of constitutive leak currents can also modulate GC bursting, and this mechanism has been shown to mediate changes in bursting during the circadian cycle (Gonzalez et al. 2023). GC action potentials exhibit a large, slow post‐burst after‐hyperpolarization. K_v_7 and K_ir_6 channels shape this after‐hyperpolarization and control bursting (Laker et al. 2021). The prolongation of inter‐spike intervals by gabazine indicates that inhibition influences the time window for repetitive firing, and shunting inhibition by unliganded opening or tonically activated GABA_A_R receptors (Wlodarczyk et al. 2013) could shorten this time window (Tang et al. 2011). A reduction in GABA_A_ receptor‐mediated shunting inhibition may contribute to the higher excitability of GCs recruited during behavior (Pléau et al. 2020). Thus, bursting is controlled by many molecules capable of contributing to the adaptive changes in GC firing reported here. The generation of doublets and the distribution of intervals may reflect distinct gene expression profiles that determine the functional state of a GC.

Changes in incidence and interval of doublets could be shaped not only by intrinsic excitability and inhibition, but also by experience‐dependent changes in synaptic connectivity. Recent work using Fos‐based engram cell labeling in combination with dual‐eGRASP to visualize four types of synapses formed between engram and non‐engram cells showed that the density and size of engram‐cell‐to‐engram‐cell synapses in the CA3–CA1 circuit increased selectively in proportion to memory strength, even though the number of engram cells remained constant (Choi et al. 2018). This finding suggests that connectivity between engram cells contributes to the encoding. Prior studies in the DG across distinct behavioral paradigms have also shown activity‐dependent increases in synapse number, spine density, and synaptic strength (Chatzi et al. 2019; Ryan et al. 2015). This form of synaptic rearrangement can enhance excitatory drive from the PP and mossy cell inputs to increase burst generation. Although our study does not directly assess excitatory connectivity, the emergence of more frequent bursts in experience‐related TRAP‐GCs may reflect both synaptic strengthening and circuit‐level rewiring that enhances excitability and facilitates activation in behaviorally relevant contexts.

GC axons have three different postsynaptic targets: CA3 pyramidal cells, hilar mossy cells, and inhibitory interneurons (Acsady et al. 1998; Toth et al. 2000). Transmission to each has a distinct form of use‐dependent plasticity (Geiger and Jonas 2000; Hashimotodani et al. 2017; Henze et al. 2002; McBain 2008; Mori et al. 2004; Mori et al. 2007). Bursts facilitate transmission, and variations in their duration and frequency will lead to preferential facilitation among these different targets to select the pathway for information flow through the hippocampus (Dumenieu et al. 2018; Henze et al. 2002; Neubrandt et al. 2018). Increasing burst incidence without changing the inter‐spike interval will enhance transmission to current targets, while changes in the inter‐spike interval will redirect activity between targets. Repetitive spiking constitutes a form of temporal coding fundamental to the processing of information, with burst properties adapted to produce an output appropriate to a specific sensory input. The incidence and interval of repetitive firing in GCs thus represent two distinct elements of the code for processing information, both of which can be shaped by experience to modify behavior.

## Conflicts of Interest

The authors declare no conflicts of interest.

## Data Availability

The data that support the findings of this study are available from the corresponding author upon reasonable request.
